# Intestinal Obstruction following Ingestion of Metallic Instruments in a Psychiatric Patient

**DOI:** 10.1155/2018/2469462

**Published:** 2018-10-17

**Authors:** Oscar Kivike, Israel Soko, David Mgaya, Frank Sandi

**Affiliations:** ^1^Department of Surgery, College of Health Sciences, The University of Dodoma, Tanzania; ^2^The University of Dodoma, Tanzania; ^3^Muhimbili University College of Health, Tanzania

## Abstract

Pica among psychiatric patients has been well documented. We report a 25-year-old female patient who presented with abdominal distension for one week. She is a known psychiatric patient for 5 years. Through history taking, physical examination, and investigations, the patient was found to have psychotic features and features of intestinal obstruction. Surgery was done by opening the abdomen and then the stomach. The stomach, together with the proximal intestine, was found to be filled with metallic instruments weighing 780 mg. The diagnosis of a metalophagia type of pica was reached. All instruments were removed and the patient did well postoperatively.

## 1. Introduction

Pica is defined by the American Psychiatric Association as the behavior of eating nonfood/nonnutritive substances consistently for over a month that is not culturally approved [1]. What causes pica remains not clearly defined, and it remains a debatable topic. Several hypotheses have tried to link pica with nutritional, psychological, cultural, and pharmacological deficits and disease. The disorder has been reported to be common among pregnant women [2], individuals with developmental disabilities, and psychiatric patients.

Pica may take different forms depending on the substance consumed; these include pagophagia (eating ice), trichophagia (eating hair), xylophagia (eating paper), lithophagia (eating stones), geophagia (eating soil), and metallophagia (eating metals). In literature, geophagia and pagophagia have been well documented both being linked with iron deficiency anaemia [3]. Other forms are not well documented in literature.

## 2. Case Report

**Figure 1:**
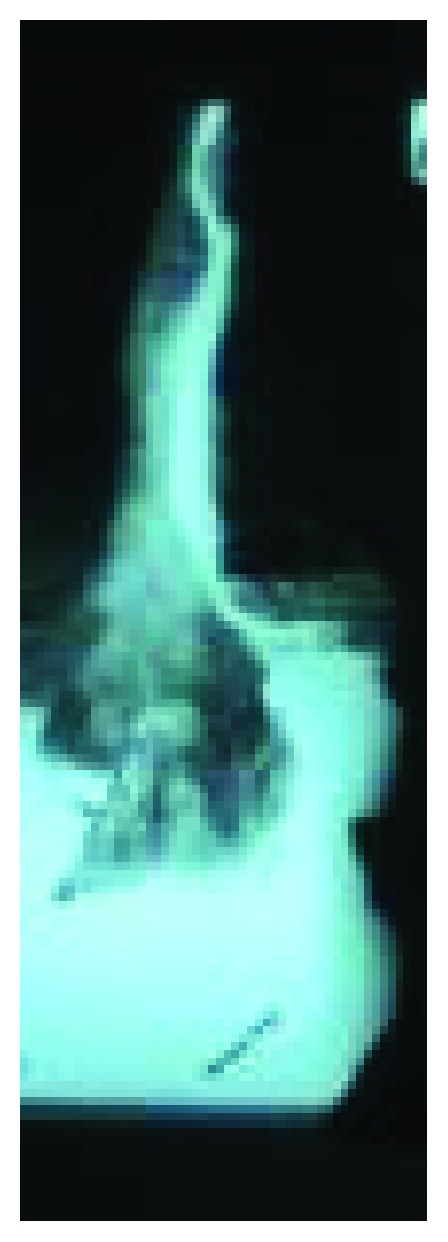
Barium meal X-ray showing stomach extending to the pelvis.

**Figure 2:**
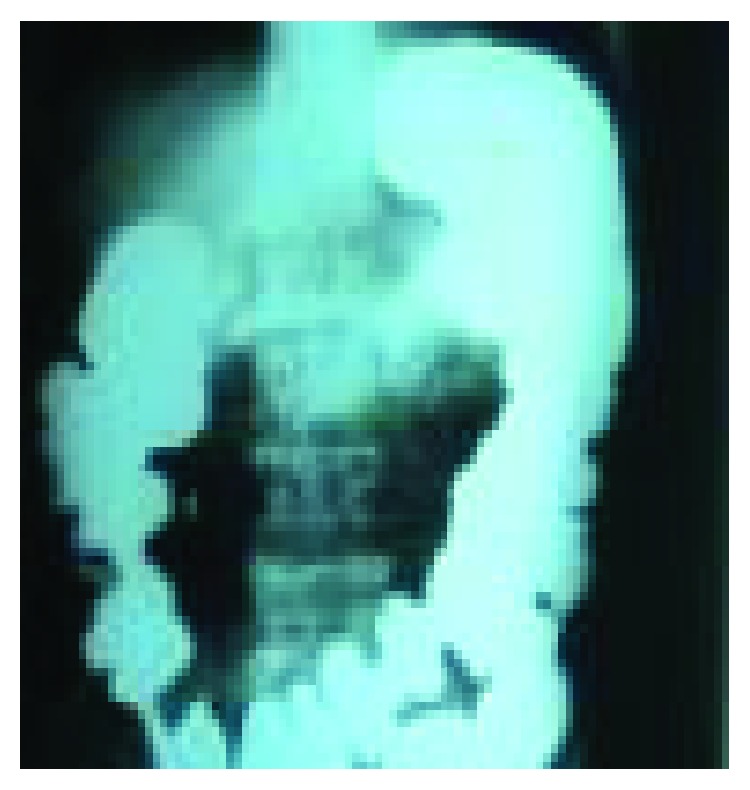
Barium meal X-ray showing stenosis and irregularities in some parts of the intestine.

We report the case of a 25-year-old female patient with a long-standing history of psychiatric disorder on medical treatment who was referred to our unit from a health centre with a history of abdominal pain, abdominal distension, and failure to pass stool and flatus for one week. On examination, the patient had disorganized speech, abnormal motor behavior, and lack of emotional expression. The abdomen was distended to below the umbilicus, irregular hard multiple masses were palpable below the umbilicus, and bowel sounds were found to be exaggerated. Abdominal pelvic ultrasound was done and revealed abnormal materials in the abdominal cavity. Barium meal X-ray was done and showed the stomach being located in the pelvic brim. It also showed stenosis in some parts of the gastrointestinal tract and irregularities in other parts (Figures 1 and 2).

### 2.1. Management

**Figure 3:**
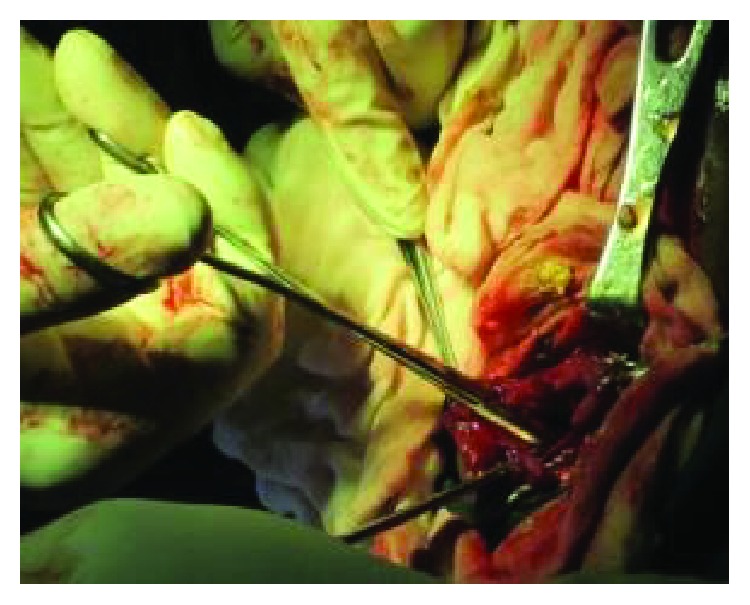
Gastrotomy and retrieval of metallic objects.

Based on the clinical presentation and X-ray findings, a decision to operate was reached. Laparotomy was done, and the stomach was found to be distended reaching the pelvis. Gastrotomy was then performed (Figure 3).

### 2.2. Findings

**Figure 4:**
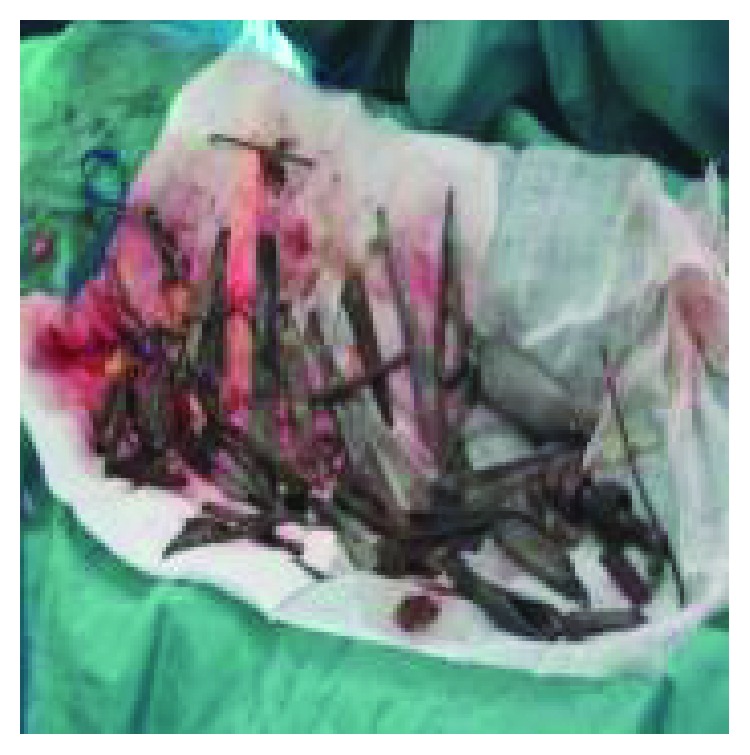
Nails extracted from the intestine of the patient.

**Figure 5:**
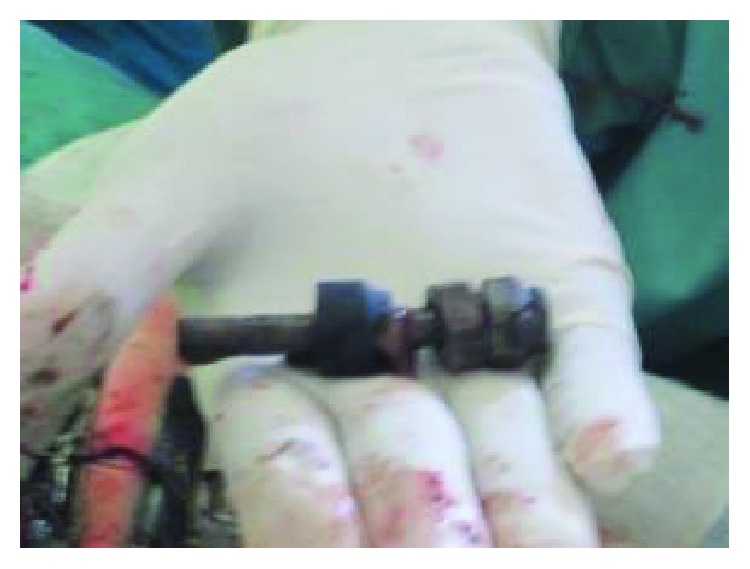
A bolt extracted from the patient.

**Figure 6:**
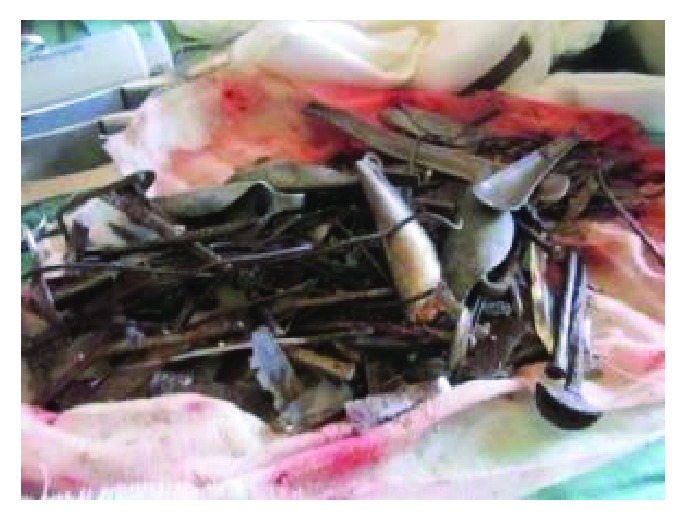
Various metallic objects retrieved from the patient.

**Figure 7:**
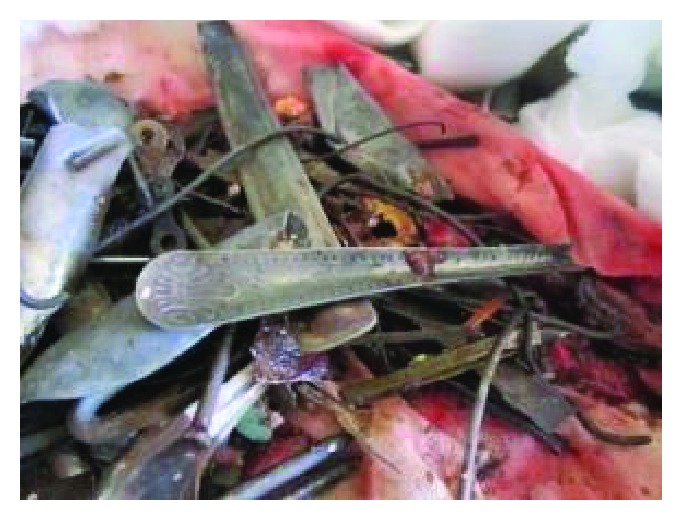
Various metallic objects retrieved from the patient.

**Figure 8:**
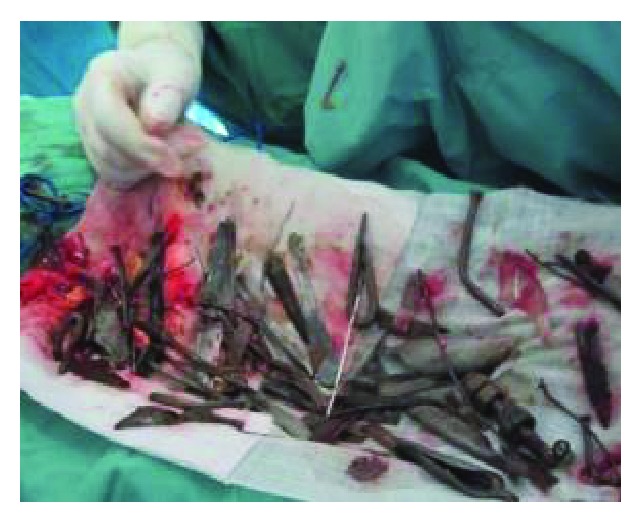
Various metallic objects retrieved from the patient.

Different metallic and nonmetallic materials were found in the stomach and proximal part of the small intestine. They were both carefully retrieved. The instruments were of various sizes and included iron nails, arrows, wheel spokes, dinner forks, broken handles of spoons with sharp edges, and many other objects weighing a total of 780 mg (Figures 4–8). The longest instrument was found to be approximately 80 mm long, and it was a nail. There was no evidence of either perforation or ulceration of both the stomach and proximal bowel. The final diagnosis of metalophagia was reached. The patient recovered and did well a few days postoperatively and was referred back to the psychiatric hospital.

## 3. Discussion

Pica is defined in the Diagnostic and Statistical Manual of Mental Disorders as the persistent habit of eating nonnutritive substances lasting for over a month; it should be severe enough to warrant clinical assessment [1]. The case we have reported presented with features of intestinal obstruction that necessitated laparotomy. We extracted metallic objects in our patient and the working diagnosis became metalophagia; one of the forms of pica.

Metalophagia is one of the forms of pica in which a person persistently consumes metallic objects. Metal eating in adults is rare; there have been very few cases reported globally on metal-eating disorder. In 2007, a case was reported in Nigeria of a 22-year-old male who presented with vomiting after meals [4]. Imaging studies revealed metallic objects in the upper part of the abdomen. Surgery was done and a total of 497 metallic objects were retrieved weighing a total of 184 kg. In our case, the metallic objects had a total weight of 780 g. Similar to our case, despite such a huge amount of metallic objects, some of which were sharp, the stomach was found to be intact.

Another reported case of metal eating was of a 2-year-old girl who started the habit at the age of 18 months [5]. She presented with a history of poor appetite and declining growth centiles. This child was later found to have zinc deficiency; she was given zinc supplements and the pica rapidly disappeared. We are not sure whether zinc deficiency was also a contributing factor for our patient; we could not measure zinc levels due to the limitations in our facility.

Most of the ingested substances into the gastrointestinal tract pass through the rectum asymptomatically; however, sharp and other metallic objects like those ingested by our patient may not be able to pass through. As a result, a patient may present with some complications like impaction which may then lead to intestinal obstruction, ulceration, gastrointestinal perforation, and bleeding, all of which may necessitate surgical intervention. Surprisingly in our patient, despite ingesting such a huge amount of metallic objects, some of which were sharp, she presented with an intact stomach and intestine with only impaction being the complication.

Despite being characterized as one of the forms of pica, reports on metal-eating disorder (metalophagia) are scarce. There are very few. In fact, only a couple of cases are documented in literature on the subject. Our case adds to the existing body of literature on this form of pica. Our patient is a known psychiatric patient; this has been documented in the literature as one of the causes of pica.

## 4. Conclusion

Despite being a rare disorder in adults, metal-eating disorder has been reported in psychiatric individuals. More evidence is needed to further understand this disorder.
